# Special Issue “Inflammatory Airway Diseases: Diagnosis, Pathology, Molecular Mechanisms and Treatment Options”

**DOI:** 10.3390/ijms27042057

**Published:** 2026-02-22

**Authors:** Emma Matthews, Stefanie Krick

**Affiliations:** 1 Division of Pulmonary, Allergy, and Critical Care Medicine, Department of Medicine, The University of Alabama at Birmingham, Birmingham, AL 35294, USA; elmatthe@uab.edu; 2 Gregory Fleming James Cystic Fibrosis Research Center, The University of Alabama at Birmingham, Birmingham, AL 35294, USA

The lung is a unique organ because of its continuous exposure to the external environment, which requires a complex and tightly regulated immune network. To preserve health and homeostasis, this network facilitates appropriate inflammatory responses to protect against harmful external stimuli, such as pathogens and pollutants. [1]. However, when these mechanisms malfunction, they can have a pathological role in diseases characterized by either acute or chronic pulmonary inflammation. The pathobiology of pulmonary diseases such as cystic fibrosis, asthma, acute respiratory distress syndrome, and interstitial lung disease all show activation of pro-inflammatory signaling pathways leading to pulmonary injury [2,3,4,5,6]. These diseases can be challenging to diagnose due to overlap in presentation and pathobiology, but early diagnosis is crucial, especially given that most diseases only have treatment options available that are not curative but help slow down disease progression. Therefore, it is important to highlight recent novel insights in the diagnosis, pathology, molecular mechanisms, and treatment options of inflammatory airway diseases to inform further research.

This Special Issue is composed of two review articles and five original research articles, expanding collective knowledge on the current field of inflammatory airway disease. Below we provide a brief statement of each manuscript included in this Special Issue.

Valladares et al. compiled a comprehensive review synthesizing current research and concluded that the burden of CF lung pathogens generally decreases following highly effective modulator therapy, but that *Staphylococcus aureus* rates overtake those of *Pseudomonas aeruginosa* as the dominant pulmonary organism (contribution 1). Malaya et al. analyzed the currently published clinical data on patients who have a rare form of severe asthma, characterized by type 2 inflammation and hypereosinophilia, to highlight the variable diagnostic criteria and lack of effective treatment options for many of these patients (contribution 2).

In multiple original research articles featured in this Special Issue, the authors explored ways to improve diagnostics and knowledge on disease pathology in human samples. Fuchs et al. (contribution 3) measured cytokine levels in nasal lavage and induced sputum samples from patients with either mild or severe cystic fibrosis lung disease and observed increased cytokines in sputum samples from patients with severe lung disease. However, patients with mild lung disease showed the highest cytokine levels in nasal lavage samples, suggesting a more pronounced immune activation in the nasal mucosa. This finding highlights the importance of sampling site when interpreting immune responses using specific collection methods. Mustafina et al. (contribution 4) sought to improve non-invasive diagnostics for lymphangioleiomyomatosis, a rare progressive lung disease, as the current diagnostic methods are costly or not specific enough. Using real-time proton mass spectrometry, they identified several potential biomarkers associated with the disease and disease severity in the volatile organic compounds of exhaled breath condensate samples from patients with lymphangioleiomyomatosis when compared to controls. Lenarčič et al. (contribution 5) sought to enhance diagnostic approaches for immune-mediated airway diseases such as hypersensitivity pneumonitis and sarcoidosis through cytokine profiling of bronchoalveolar lavage samples. This approach revealed nine potential biomarkers for these diseases, two of which were also associated with worsened pulmonary fibrosis.

Two original research articles in this Special Issue aimed to investigate the underlying molecular mechanisms of potential therapeutics for inflammatory lung diseases. Woo et al. (contribution 6) explored the effect of Broncho-Vaxom, a product containing lyophilized respiratory pathogen components, in a mouse model of acute lung injury. They found pre-treatment with Broncho-Vaxom significantly reduced the pro-inflammatory response via the NF- κB pathway, indicating it as a potential treatment for pulmonary inflammation in acute lung injury. Peng et al. (contribution 7) sought to determine the effect of hyaluronan at different molecular weights on a rat model of acute respiratory distress syndrome, an often-fatal inflammatory lung disease with few effective treatments. Their findings indicated that hyaluronan with different molecular weights had distinct therapeutic effects, and that treatment with mixed molecular weight hyaluronan yielded the strongest synergistic improvement in lung structure and function.

We hope that this Special Issue serves as a timely informative resource to vitalize future discoveries in the management of inflammatory airway diseases. We thank all of the authors, the reviewers, and the staff of the *International Journal of Molecular Sciences* for their support in creating this Editorial.
